# The E3 ligase β-TRCP1 earmarks OTUD3 for destruction to fine-tune cGAS activation

**DOI:** 10.1016/j.jbc.2026.111461

**Published:** 2026-04-17

**Authors:** Jianfeng Chen, Smaran Sivashankar, Ying Wang, Pengda Liu

**Affiliations:** 1 Lineberger Comprehensive Cancer Center, The University of North Carolina at Chapel Hill, Chapel Hill, North Carolina, USA; 2 Department of Biochemistry and Biophysics, The University of North Carolina at Chapel Hill, Chapel Hill, North Carolina, USA

**Keywords:** β-TRCP1, cGAS, innate immunity, OTUD3, RSK3, protein stability

## Abstract

Activation of cytosolic DNA sensing through cyclic GMP-AMP synthase (cGAS) induces the production of type I interferons and proinflammatory cytokines, which are essential for antiviral and antibacterial responses, inflammation, and immune modulation. While hyperactivation of cGAS leads to autoimmune diseases, its inactivation contributes to immune evasion and resistance to immunotherapies. Therefore, cGAS activity must be tightly regulated. One mechanism involves the deubiquitination and stabilization of cGAS by the deubiquitinase OTUD3; however, the upstream signals and pathophysiological cues governing OTUD3 regulation remain poorly understood. Here, we report that the E3 ubiquitin ligase β-TRCP1 targets OTUD3 for ubiquitination and proteasomal degradation. This recognition is dependent on RSK3-mediated phosphorylation of a conserved “ESG” motif in OTUD3, which serves as a phospho-degron for β-TRCP1 binding. Intriguingly, cytosolic DNA challenge inactivates the β-TRCP1/RSK3 pathway, resulting in OTUD3 stabilization and enhanced cGAS activation, representing a fine-tuning mechanism of innate immune signaling. Notably, this DNA-induced inactivation of RSK3 is independent of canonical Ras/MEK/extracellular signal-regulated kinase signaling and DNA damage-responsive kinases, but dependent on mTORC2 signaling. Collectively, our studies identify β-TRCP1/RSK3 as a previously unrecognized upstream signaling axis that regulates OTUD3 protein stability in response to DNA stress, thereby modulating cGAS-driven innate immune responses. This pathway presents a potential therapeutic target for modulating innate immunity in autoimmune diseases and cancer.

The innate immune response, broadly active across most cell types, serves as the first line of defense against invading pathogens in mammals. Among various stimuli that activate innate immunity, cytosolic nucleic acids, including both DNA and RNA, represent a distinct class of danger signals (1). Cytosolic DNA can originate from exogenous sources such as viral or bacterial infections, or from endogenous sources including nuclear or mitochondrial DNA leakage due to cellular damage (2). Activation of cytosolic DNA sensing signaling induces the production of type I interferons (IFNs), pro-inflammatory cytokines, and chemokines, which mediate antiviral and antibacterial responses, inflammation, and antitumor immune modulation (3). Numerous cytosolic DNA sensors have been proposed, but two have been established as major players: cyclic GMP-AMP synthase (cGAS) (4), which triggers IFN-β secretion (3), and absent in melanoma 2 (5, 6, 7), which activates the inflammasome (8). Absent in melanoma 2 recognizes DNA in specific cell types (9, 10), whereas cGAS binds cytosolic dsDNA in a sequence-independent but length-dependent manner (11) across most cell types. Hyperactivation of cGAS signaling is linked to autoimmune diseases (12, 13), while impaired DNA sensing allows tumors to evade immune surveillance and contributes to resistance against cancer immunotherapies (14). Consequently, cGAS activation is tightly regulated by post-translational modifications (15), interacting proteins (3), and other mechanisms under various pathophysiological conditions. For instance, cGAS can be inactivated by phosphorylation through Akt (16), CDK1 (17), mitotic kinases (18) or mTORC2 (19), and activated by ubiquitination mediated by TRIM56 (20) or RNF185 (21). Moreover, deubiquitination of cGAS by USP14 (22), USP27X (23), and OTUD3 (24, 25) stabilizes the protein by removing K48-linked polyubiquitin chains.

Protein ubiquitination plays critical roles in regulating various aspects of protein function, including stability, trafficking, binding interactions, and cellular localization (26). This modification is reversible: polyubiquitin chains, added by E3 ligases, can be removed by deubiquitinases (DUBs). Both E3 ligases and DUBs are structurally and functionally diverse, with distinct mechanistic properties. While more than 600 E3 ligases exist, only about 100 DUBs have been identified, suggesting that DUBs may be less selective toward substrates. DUBs possess either cysteine protease or metalloprotease activity and are classified into seven families: ubiquitin-specific proteases (USPs), ovarian tumor protease (OTUs), ubiquitin C-terminal hydrolases, Josephins (Machado-Josephin domain proteases), MIU-containing novel DUB family, Jab1/Mov34/Mpr1 Pad1 N-terminal proteases, and zinc finger with UFM1-specific peptidases (27, 28, 29). Despite their structural diversity, all DUBs recognize a common hydrophobic patch on ubiquitin. Among these, OTU family members selectively recognize specific ubiquitin chain linkages to regulate distinct signaling pathways (30). To date, 16 mammalian OTUs have been identified, although their physiological roles are only beginning to be understood (31). We and others have reported that OTUD7B maintains mTOR complex homeostasis (32), activates NF-κB signaling (33), and regulates cell cycle progression by antagonizing APC/Cdh1 function (34). Recently, we developed the first catalytic inhibitor of OTUD7B, which exhibits anti-cancer effects (35). In addition, we recently discovered that OTUD7A regulates the protein stability of the Ewing sarcoma breakpoint region 1::Friend leukemia integration 1 oncofusion protein in Ewing sarcoma, identifying it as a novel drug target. We have also identified lead small-molecule inhibitors that target OTUD7A’s catalytic activity (36). However, context-dependent roles of OTU family DUBs have also been observed. For instance, OTUB1 promotes prostate tumorigenesis by activating RhoA (37), yet suppresses osteosarcoma cell proliferation by stabilizing p53 (38). Similarly, OTUD3 exhibits dual functions: it stabilizes PTEN (39) and p53 (40) to suppress breast tumorigenesis, but also promotes lung cancer growth by stabilizing GRP78 (41), highlighting its context-dependent activity. In innate immune signaling, OTUD3 plays opposing roles in RNA and DNA sensing pathways. It inhibits RNA sensing by removing K63-linked ubiquitin chains from mitochondrial antiviral-signaling protein, thereby blocking mitochondrial antiviral-signaling protein activation (42), while enhancing DNA sensing by deubiquitinating K48-linked polyubiquitin chains from cGAS, leading to cGAS stabilization (24). However, the mechanism by which OTUD3 distinguishes between these distinct substrates and the pathophysiological cues that govern its substrate specificity and regulatory function remain largely unknown.

In this study, we report that OTUD3 protein stability is regulated by the E3 ligase β-TRCP1, which targets OTUD3 for ubiquitination and subsequent degradation. Recognition of OTUD3 by β-TRCP1 is mediated through RSK3-dependent phosphorylation of a canonical “ESG” degron motif. Intriguingly, upon dsDNA challenge, OTUD3 is stabilized due to dsDNA-induced inactivation of RSK3, mediated *via* a previously unrecognized mTORC2 signaling, resulting in enhanced cGAS activation. This represents a fine-tuning mechanism to amplify innate immune responses. Collectively, our findings reveal an additional regulatory layer of cGAS activation mediated by OTUD3 stabilization in response to cytosolic DNA.

## Results

### The E3 ligase β-TRCP1 targets OTUD3 for ubiquitination and degradation

**Figure 1:**
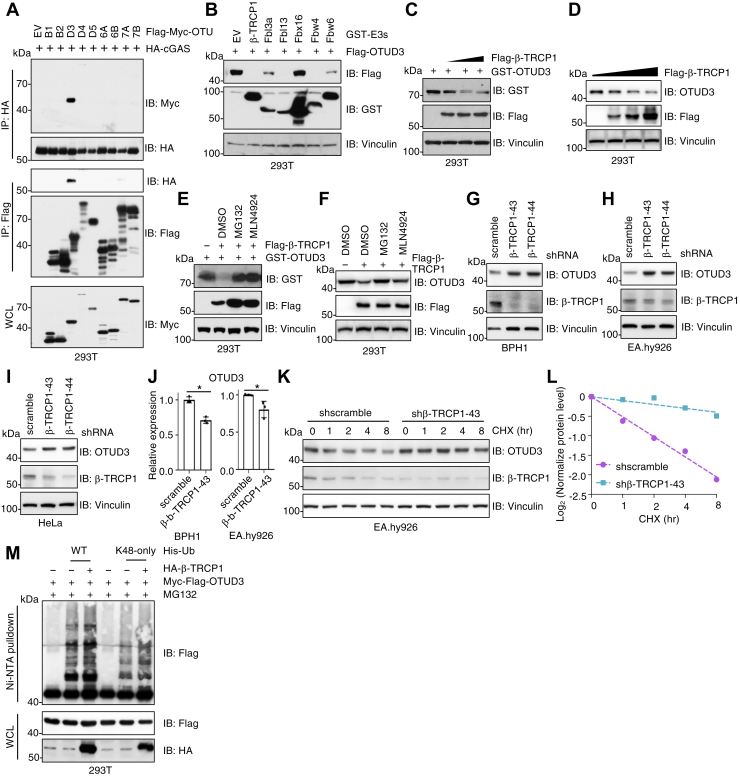
**β-TRCP1 targets OTUD3 for ubiquitination and degradation.***A*, IB analysis of HA-IPs, Flag-IPs and WCL from HEK293T cells transfected with indicated DNA constructs. *B*–*D*, IB analysis of WCL from HEK293T cells transfected with indicated DNA constructs. *E* and *F*, IB analysis of WCL from HEK293T cells transfected with indicated DNA constructs. Where indicated, 10 μM MG132 or 1 μM MLN4924 was used to treat cells for 12 h before collection. *G*–*I*, IB analysis of WCL from indicated cells. Where indicated, cells were infected with lentiviruses targeting scramble or endogenous β-TRCP1 and selected in DMEM medium containing 1 μg/ml puromycin for 72 h to eliminate non-infected cells. *J*, qRT-PCR analysis of OTUD3 mRNA expression level from indicated cells. Error bars represent SD, ∗*p*< 0.05 (two-tailed *t* test), n = 3. *K*, IB analysis of WCL from indicated EA.hy926 cells treated with 200 μg/ml cycloheximide for indicated time periods, with quantification shown in *L*. *M*, IB analysis of Ni-NTA pulldowns and WCL from HEK293T cells transfected with indicated DNA constructs. Cells were treated with 10 μM MG132 for 12 h prior to cell collection. qRT-PCR, quantitative real-time PCR; WCL, whole cell lysates; IB, immunoblot.

Consistent with previous reports (24, 25), a survey of OTU family deubiquitinases revealed that only OTUD3 specifically interacted with cGAS in cells (Fig. 1*A*). To identify potential E3 ubiquitin ligases that regulate OTUD3 protein stability, we screened a panel of F-box E3 ligases available in our lab (43) and found that β-TRCP1, Fbxl13, and Fbw4 promoted the degradation of ectopically expressed OTUD3 (Fig. 1*B*). Among them, β-TRCP1 degraded either exogenous (Fig. 1*C*) or endogenous OTUD3 (Fig. 1*D*) in a dose-dependent manner. This degradation was largely blocked by either the proteasome inhibitor MG132, or cullin neddylation inhibitor MLN4924, which is essential for F- box E3 ligase activation (Fig. 1, *E* and *F*). These data suggested that β-TRCP1 may function as an E3 ligase controlling OTUD3 protein abundance. Supporting this hypothesis, depletion of endogenous β-TRCP1 led to increased OTUD3 protein levels in BPH1 (Fig. 1*G*), EA.hy926 (Fig. 1*H*), and HeLa cells (Fig. 1*I*). Importantly, this increased OTUD3 protein was not due to elevated mRNA levels (Fig. 1*J*), indicating a post-transcriptional regulation. Furthermore, β-TRCP1 knockdown significantly extended the half-life of endogenous OTUD3 protein in BPH1 cells (Fig. 1, *K* and *L*). In line with β-TRCP1 acting as an E3 ligase, β-TRCP1 overexpression enhanced OTUD3 ubiquitination in cells, especially at K48-linked ubiquitination (Fig. 1*M*). Together, these findings suggest that β-TRCP1 might be a *bona fide* E3 ubiquitin ligase responsible for OTUD3 ubiquitination and degradation.

### β-TRCP1 recognizes a conserved “ESG” degron in OTUD3

**Figure 2:**
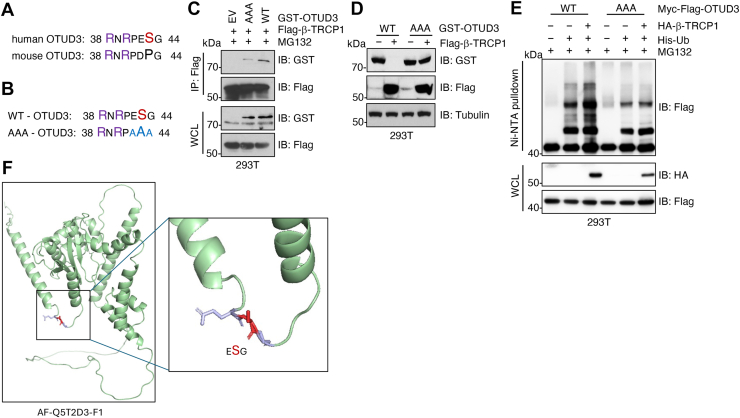
**β-TRCP1 recognizes a OTUD3-ESG degron.***A* and *B*, protein sequence of human or mouse OTUD3 ESG degrons and corresponding alanine replacement mutations. *C*, IB analysis of Flag-IPs and WCL from HEK293T cells transfected with indicated DNA constructs. Cells were treated with 10 μM MG132 for 12 h prior to cell collection. *D*, IB analysis of WCL from HEK293T cells transfected with indicated DNA constructs. *E*, IB analysis of Ni-NTA pulldowns and WCL from HEK293T cells transfected with indicated DNA constructs. Cells were treated with 10 μM MG132 for 12 h prior to cell collection. *F*, a representative image for an alpha-fold predicted OTUD3 protein structure with the ESG degron accessible on the protein surface. WCL, whole cell lysates; IB, immunoblot.

β-TRCP1 is known to recognize a conserved degron motif composed of a “D/E-S-G” sequence (43). Upon examining the OTUD3 protein sequence, we identified a conserved “D/E-S-G” motif at residues 42 to 44 in both human and mouse OTUD3 (Fig. 2*A*). Mutation of this motif to triple alanines (AAA) (Fig. 2*B*) markedly reduced OTUD3 binding to β-TRCP1 in cells (Fig. 2*C*), rendering the mutant resistant to β-TRCP1-mediated degradation (Fig. 2*D*). Consequently, compared with WT OTUD3, the AAA mutant exhibited impaired ubiquitination by β-TRCP1 in cells (Fig. 2*E*). Notably, structural analysis revealed that the “ESG” degron is surface-exposed on the OTUD3 protein (Fig. 2*F*), supporting its accessibility for recognition by β-TRCP1.

### RSK3 phosphorylates OTUD3 in the “ESG” degron for β-TRCP1 recognition

**Figure 3:**
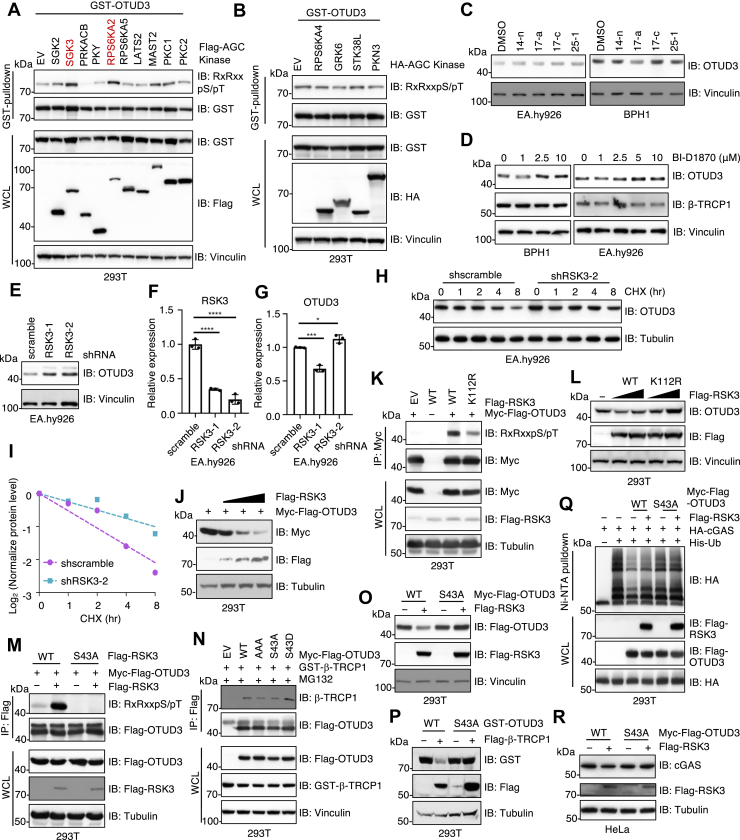
**RSK3 phosphorylates OTUD3-ESGmotifto generate a phospho-degron for β-TRCP1 recognition.***A* and *B*, IB analysis of GST-pulldowns and WCL from HEK293T cells transfected with GST-OTUD3 and indicated AGC kinase constructs. *C*, IB analysis of WCL from indicated cells treated with indicated SGK3 inhibitors at 10 μM for 12 h. *D*, IB analysis of WCL from indicated cells treated with indicated doses of BI-D1870 for 12 h. *E*, IB analysis of WCL from indicated cells. Where indicated, cells were infected with lentiviruses targeting scramble or endogenous RSK3 and selected in culture media containing 1 μg/ml puromycin for 72 h to eliminate noninfected cells. *F* and *G*, qRT-PCR analysis of indicated mRNA levels from indicated cells. *Error bars* represent SD, ∗*p*< 0.05 (one-way ANOVA), n = 3. *H*, IB analysis of WCL from indicated EA.hy926 cells treated with 200 μg/ml cycloheximide for indicated time periods, with quantification shown in *I*. *J*, IB analysis of WCL from HEK293T cells transfected with indicated DNA constructs. *K*, IB analysis of Myc-IPs and WCL from HEK293T cells transfected with indicated DNA constructs. *L*, IB analysis of WCL from HEK293T cells transfected with indicated DNA constructs. *M*, IB analysis of Flag-IPs and WCL from HEK293T cells transfected with indicated DNA constructs. *N*, IB analysis of Flag-IPs and WCL from HEK293T cells transfected with indicated DNA constructs. Cells were treated with 10 μM MG132 for 12 h prior to cell collection. *O* and *P*, IB analysis of WCL from HEK293T cells transfected with indicated DNA constructs. *Q*, IB analysis of Ni-NTA pulldowns and WCL from HEK293T cells transfected with indicated DNA constructs. *R*, IB analysis of WCL from HeLa cells transfected with indicated DNA constructs. AGC, protein kinase A, G and C families; IB, immunoblot; qRT-PCR, quantitative real-time PCR; WCL, whole cell lysates.

Most β-TRCP1 substrates require phosphorylation within the “D/E-S-G” degron motif (44), and intriguingly the OTUD3 “ESG” degron resides within an “RxRxxS/T” motif, a canonical recognition site for protein kinase A, G and C families (AGC) kinases (45). Therefore, we hypothesized that an AGC kinase might phosphorylate this motif to create a phospho-degron for β-TRCP1 recognition. To identify potential upstream kinases, we performed an AGC kinase screening using a cDNA library previously generated in our lab (46). Monitoring phosphorylation of OTUD3 at the “RxRxxS/T” motif, we found that both SGK3 and RPS6KA2 (RSK3) promoted OTUD3 phosphorylation in cells (Fig. 3, *A* and *B*). Pharmacological inhibition of SGK3 using multiple inhibitors (47) had no significant effect on OTUD3 protein levels (Fig. 3*C*). In contrast, inhibition of RSK3 using RSK inhibitor BI-D1870 led to increased OTUD3 protein levels in BPH1 and EA.hy926 cells (Fig. 3*D*), suggesting that RSK3-mediated phosphorylation may regulate OTUD3 stability. Supporting this idea, genetic depletion of RSK3 also elevated OTUD3 protein levels (Fig. 3, *E* and *F*), without affecting OTUD3 mRNA levels (Fig. 3 *G*). Similar to β-TRCP1 depletion (Fig. 1, *K* and *L*), RSK3 knockdown significantly extended OTUD3 protein half-life in cells (Fig. 3, *H* and *I*). Moreover, ectopic expression of RSK3 promoted OTUD3 degradation in a dose-dependent manner (Fig. 3*J*). Mechanistically, a catalytically inactive RSK3 mutant (K112R) displayed a reduced ability to phosphorylate OTUD3 (Fig. 3*K*), and subsequent inability in degrading endogenous OTUD3 (Fig. 3*L*). An OTUD3-S43A mutant was deficient in RSK3-mediated phosphorylation (Fig. 3*M*). Correspondingly, the phospho-deficient OTUD3-S43A mutant showed reduced binding to β-TRCP1, while the phospho-mimetic S43D mutant exhibited enhanced β-TRCP1 binding (Fig. 3*N*). As a result, both RSK3 (Fig. 3*O*) and β-TRCP1 (Fig. 3*P*) efficiently degraded OTUD3-WT but failed to degrade the OTUD3-S43A mutant. Together, these data demonstrate that RSK3 phosphorylates OTUD3 at “ESG”, creating a phospho-degron necessary for β-TRCP1 recognition and subsequent OTUD3 degradation. As a result, RSK3 partially suppresses OTUD3-mediated cGAS deubiquitination through phosphorylation of OTUD3 at S43 (Fig. 3*Q*), thereby modulating OTUD3-mediated cGAS stability (Fig. 3*R*).

### DNA challenge induces OTUD3 protein stabilization by inactivating RSK3 to enhance cGAS activation

**Figure 4:**
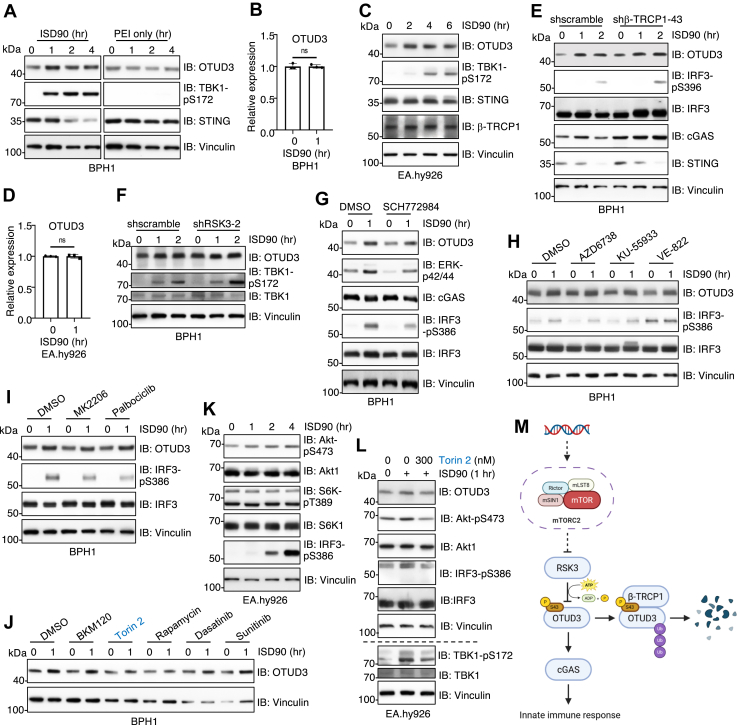
**dsDNA-mediated inactivation of RSK3 stabilizes OTUD3 to promote cGAS activation.** AuthorAnonymous, *A* and *C*, IB analysis of WCL from BPH1 (*A*) or EA.hy926 cells (*C*) transfected with 5 μg/ml ISD90 or polyethylenimine only for indicated time periods. *B* and *D*, qRT-PCR analysis of OTUD3 mRNAs from indicated cells. Error bars represent SD, ∗*p*< 0.05 (two-tailed *t* test), n = 2. *E* and *F*, IB analysis of WCL from indicated BPH1 cells transfected with 5 μg/ml ISD90 for indicated time periods. *G*, IB analysis of WCL from BPH1 cells transfected with 5 μg/ml ISD90 for indicated time periods. Where indicated, cells were pre-treated with 2 μM ERK inhibitor SCH772984 for 1 h. *H*, IB analysis of WCL from BPH1 cells transfected with 5 μg/ml ISD90 for the indicated time points. Where specified, cells were pre-treated with indicated inhibitors at 2 μM for 1 h prior to transfection. *I*, IB analysis of WCL from BPH1 cells transfected with 5 μg/ml ISD90 for the indicated time points. Where specified, cells were pretreated with 100 nM MK2206 or 10 μM palbociclib for 1 h prior to transfection. *J*, IB analysis of WCL from BPH1 cells transfected with 5 μg/ml ISD90 for the indicated time points. Where specified, cells were pre-treated with BKM120 (500 nM), Torin 2 (100 nM), Rapamycin (50 nM), Dasatinib (1 μM), or Sunitinib (10 μM) for 1 h prior to transfection. *K* and *L*, IB analysis of WCL from EA.hy926 cells transfected with 5 μg/ml ISD90 for the indicated time points. Where specified, cells were pretreated with 300 nM Torin two for 1 h prior to transfection. *M*, a cartoon illustration of the proposed model generated by Biorender. ERK, extracellular signal-regulated kinase; IB, immunoblot; qRT-PCR, quantitative real-time PCR; WCL, whole cell lysates.

We next investigated the physiological relevance of the β-TRCP1/RSK3-mediated regulation of OTUD3 protein stability in cGAS-dependent innate immune activation. Upon stimulation with ISD90, a synthetic dsDNA mimic, we observed a marked increase in OTUD3 protein levels in both BPH1 (Fig. 4*A*) and EA.hy926 cells (Fig. 4*C*). Notably, OTUD3 mRNA levels remained unchanged (Fig. 4, *B* and *D*), suggesting a post-transcriptional mechanism of regulation. These findings led us to hypothesize that dsDNA challenge inactivates the β-TRCP1/RSK3 pathway, thereby stabilizing OTUD3 protein. Supporting this hypothesis, depletion of either β-TRCP1 (Fig. 4*E*) or RSK3 (Fig. 4*F*) attenuated ISD90-induced OTUD3 protein accumulation. Furthermore, in line with previous studies (24, 25), stabilized OTUD3 resulting from β-TRCP1 or RSK3 depletion significantly enhanced cGAS pathway activation, evidenced by increased p-IRF3 or pTBK1 signals following ISD90 stimulation (Fig. 4, *E* and *F*). These results support a model in which the β-TRCP1/RSK3 axis serves as a negative regulator of OTUD3 protein stability, functioning as a fine-tuning mechanism to modulate cGAS activity during innate immune responses.

Interestingly, these findings also suggest that dsDNA stimuli such as ISD90 may suppress β-TRCP1/RSK3 signaling to facilitate OTUD3 stabilization. Given that RSK3 is canonically activated downstream of the Ras/MEK/extracellular signal-regulated kinase (ERK) pathway (48), we tested whether this signaling axis is involved in ISD90-induced RSK3 inactivation. Treatment with the ERK inhibitor SCH772984 failed to block OTUD3 stabilization upon ISD90 stimulation (Fig. 4*G*), suggesting that Ras/MEK/ERK signaling is not responsible for dsDNA-mediated RSK3 inactivation. Since cytosolic DNA and DNA double-strand breaks are known to trigger DNA damage responses involving the activation of ATM/ATR kinases (49), we next examined whether these kinases contribute to RSK3 inactivation upon ISD90 stimulation. To this end, we utilized specific inhibitors targeting ATM and ATR kinases and found that all the inhibitors failed to block OTUD3 stabilization as well (Fig. 4*H*), indicating that OTUD3 stability was independent of ATM/ATR activation. Finally, we tested the role of a series of canonical regulatory pathways by inhibiting various key kinase targets, including PI3K (BKM120), mTOR (Torin 2), mTORC1 (Rapamycin), Akt (MK2206), RTK (Dasatinib, Sunitinib), and CDK4/6 (Palbociclib). Surprisingly, the mTORC2 pathway is activated upon dsDNA challenge and inhibition of mTORC2 signaling *via* Torin two blocked OTUD3 stability presumably through RSK3 (Fig. 4, *I*–*L*). These results show that upon dsDNA stimulus, the mTORC2 kinase is activated to suppress RSK3 activity, through an unknown mechanism(s). This trend reduces RSK3-mediated OTUD3 phosphorylation, allowing OTUD3 escaping from recognition and degradation by the E3 ligase β-TRCP1, facilitating cGAS-mediated innate immune response (Fig. 4*M*).

## Discussion

In this study, we found that OTUD3 is a substrate for the E3 ligase β-TRCP1, which recognizes RSK3-mediated phosphorylation of the conserved “ESG” motif in OTUD3 for ubiquitination and degradation. Further, we showed that the β-TRCP1/RSK3 axis is suppressed in response to dsDNA challenge and plays a crucial role in modulating OTUD3 protein levels, cGAS activation, and thereby, innate immune response. Previously, β-TRCP1 was reported to have indirect effects in regulating cGAS, serving as an E3 ligase for the protein MYO10, which drives genomic instability and indirectly activates cGAS (50). Yet, β-TRCP1 plays a role in directly regulating cellular response to stress more broadly and its response has been shown to be regulated in turn by cell stress (50). For example, β-TRCP1 degrades IκB upon its phosphorylation, which is upregulated by viral infection, and this degradation yields stress-adaptive cellular changes (51, 52, 53, 54). Our results, which demonstrate that β-TRCP1 negatively regulates OTUD3 in response to dsDNA challenge, further elucidate the role of β-TRCP1 in regulation of innate immune response upon dsDNA challenge, clarifying its important role specifically in cGAS-mediated innate immune response.

Stimulation with dsDNA increased OTUD3 protein levels, presumably through inactivation of the RSK3-mediated OTUD3 phosphorylation that would otherwise promotes degradation by β-TRCP1. Previous studies have reported opposing roles of OTUD3 in DNA *versus* RNA viral infections. RNA virus infection leads to SIRT1-dependent deacetylation of OTUD3 (42), which inactivates OTUD3 and limits activation of the RNA sensors RIG-I and MDA5 due to reduced OTUD3-mediated K63-linked ubiquitination (24). In contrast, OTUD3 has been shown to promote DNA sensing by stabilizing the cGAS protein (24) and contributing to the formation of cGAS/DNA droplets (25). However, the mechanism through which DNA viral infection regulates OTUD3 has remained unclear. Our findings suggest that DNA viral infection stabilizes OTUD3 by inhibiting RSK3-mediated phosphorylation, thereby preventing β-TRCP1-mediated ubiquitination and degradation. Consistent with earlier studies (24, 25), stabilized OTUD3 enhances cGAS activation and subsequent innate immune responses. Notably, OTUD3 stabilization occurs during the early phase of DNA stimulation, suggesting that this regulatory mechanism may serve as an early-response checkpoint to ensure timely amplification of cGAS-driven innate immune activation. Future studies examining pathophysiological roles of β-TRCP1/RSK3-mediated OTUD3 protein stability control are warranted in the context of DNA viral and bacterial infection. In addition, beyond the signaling studies included in this study, further downstream effectors of the cGAS/STING signaling such as IFN-β and subsequent ISGs would need to be monitored in additional cellular or animal models.

Several proteins which directly regulate cGAS *via* ubiquitin modulation have been revealed to possess a dsDNA-induced fine-tuning mechanism. For example, RNF185 was identified as an E3 ubiquitin ligase which improves cGAS stability and the RNF185/cGAS association is enhanced upon HSV-1 infection, augmenting cGAS activation (22). USP14, a DUB that acts on cGAS, is recruited by TRIM14 to remove K48-ubiquitin chains in cGAS, yielding cGAS stabilization and TRIM14 itself was shown to be upregulated by polydAdT transfection (22). Yet, most studies examining DUBs acting on cGAS have explored minimal evidence for mechanisms of upstream control, especially how dsDNA regulates the axis directly engaging the DUB (22, 23, 24, 25). Beyond reporting β-TRCP1 as a *bona fide* E3 ligase for OTUD3 ubiquitination and degradation, our studies further reveal that dsDNA activates mTORC2, which in turn may suppress RSK3 kinase activity. As a result, the RSK3-generated OTUD3 phospho-degron is abolished, allowing OTUD3 to escape β-TRCP1-mediated recognition and degradation. Further in-depth investigation is warranted to explore how dsDNA induces mTORC2 activation, how mTORC2 negatively regulates RSK3 activity, and whether additional mTORC2 activating stimuli, such as growth factor signaling, would similarly inactivate RSK3 to stabilize OTUD3.

## Experimental procedures

### Materials

Glutathione agarose beads (17-0756-05) were obtained from GE Healthcare. Nickel agarose beads (H-320-5) were purchased from GoldBio. Anti-Flag agarose beads (A-2220), anti-c-Myc magnetic beads (SAE0201), anti-HA agarose beads (A-2095), and puromycin (P8833) were purchased from Sigma-Aldrich. MG132 (S2619) and Cycloheximide (C7698) were purchased from Selleck. Protease inhibitor cocktail (K1008) and phosphatase inhibitor cocktail (K1015) were obtained from Apexbio Technology. BI-D1870 (HY-10510), MLN4924 (HY-70062), AZD6738 (HY-19323), KU-55933 (HY-12016), VE-822 (HY-13902), SCH772984 (HY-50846), MK2206 (HY-108232), Palbociclib (HY-50767), BKM120 (HY-70063), Torin 2 (HY-13002), Rapamycin (HY-10219), Dasatinib (HY-10181), and Sunitinib (HY-10255A) were purchased from MedChemExpress. SGK inhibitors, including 14-n, 17-a, 17-c, and 25-i, were obtained from the Robert Hagan lab at UNC Chapel Hill.

### Antibodies

All antibodies were used at a 1:2000 dilution in TBST buffer with 5% nonfat milk for western blotting. Anti-Flag antibody (F-1804) was obtained from Sigma-Aldrich. Anti-GST antibody (sc-459) was obtained from Santa Cruz Biotechnology. Anti Myc antibody (71D10), anti-phospho-Akt (Ser473) antibody (4060), anti phospho-Akt substrate (RxRxxS∗/T∗) antibody (10,001), anti-TBK1-pSer172 antibody (5483), anti-TBK1 antibody (51,872), anti-STING antibody (13,647), anti-cGAS antibody (15,102), anti-IRF3-pSer386 (37,829), anti-IRF3-pSer396 (4947), anti-IRF3 antibody (4302), anti-MAPK-p42/44 antibody (9102), anti-Akt1 antibody (2938), anti-S6K-pT389 antibody (9205), anti-S6K1 antibody (9202), and anti β-TRCP1 antibody (11,984) were obtained from Cell Signaling Technology. Anti-HA antibody (51064-2-AP), anti-tubulin antibody (66,240-1-lg), anti-vinculin antibody (26520-1-AP), and anti-OTUD3 antibody (29622-1-AP) were obtained from Proteintech.

### Cell culture and transfection

Immortalized human embryonic kidney-derived cell line HEK293T, human cervical cancer cell line HeLa, human benign prostate tumor cell line BPH1, and human somatic hybrid cell line EA.hy926 cells were cultured in DMEM medium, supplemented with 10% FBS, 100 U penicillin and 100 mg/ml streptomycin in a 37 °C incubator with 5% CO_2._ All cell lines have been closely monitored to avoid any contamination. No short tandem repeat profiling has been performed for authentication of these cell lines.

Cell transfection was performed using polyethylenimine, as described previously (36, 55, 56). Packaging of lentiviral shRNA viruses, as well as subsequent infection of various cell lines, were performed according to the protocols described previously (55, 56). Following viral infection, cells were maintained in the presence of puromycin (1 μg/ml) for selection.

### Plasmids

HA-β-TRCP1, Flag-β-TRCP1, and GST-OTUD3 vectors were constructed by inserting the relevant gene segment into pCDNA3.0-HA, pCDNA3.0-Flag, and CMV-GST vectors. S43A, S43D, and AAA related OTUD3 vectors and K112R-Flag-RSK3 were obtained using the Site-Direction Mutagenesis Kit from Agilent (200523). Flag/HA-AGC kinase constructs are as described previously (46). Flag-Myc-OTUs and GST-E3s vectors were as previously described (36, 44). WT and K48-linked His-Ub plasmids were described (57, 58). shRNA plasmids were constructed by inserting synthesized shRNAs into the pLKO-puro vector. All the primers used for vector constructions are listed below.

### OTUD3-BamH1-F: GCATGGATCCTCCCGAAAGCAGGCGGCGAA

OTUD3-Xho1-R: GCATCTCGAGTCAGATGTTGAGAGCGGCGA.

S43A-OTUD3-F: AATCGGCCGGAGGCTGGCGGCGGCGGC.

S43A-OTUD3-R: GCCGCCGCCGCCAGCCTCCGGCCGATT.

S43D-OTUD3-F: AATCGGCCGGAGGATGGCGGCGGCGGC.

S43D-OTUD3-R: GCCGCCGCCGCCATCCTCCGGCCGATT.

AAA-OTUD3-F: AATCGGCCGGCGGCTGCCGGCGGCGGC.

AAA-OTUD3-R: GCCGCCGCCGGCAGCCGCCGGCCGATT.

TRCP1-BamH1-F: GCATGGATCCGACCCGGCCGAGGCGGTGCT.

TRCP1-Xho1-R: GCATCTCGAGTTATCTGGAGATGTAGGTGT

cGAS-BglII-F: GCATAGATCTCAGCCTTGGCACGGAA

cGAS-Xho1-R: GCATCTCGAGTCAAAATTCATCAAAAA

shTRCP1-43: GCGTTGTATTCGATTTGATAA

shTRCP1-44: GCGTTTCAATAATGGCATGAT

shRSK3-1: GCAAGTTTGTGTACCTGGTAA

shRSK3-2: CGCCACCTACTTTGCTCTAAA.

### Immunoblot and immunoprecipitation analyses

Cells were lysed in EBC buffer (50 mM Tris-HCl, pH 7.5, 120 mM NaCl, 0.5% NP-40) supplemented with protease and phosphatase inhibitor cocktails. Protein concentrations of whole cell lysates were determined using the Bradford method with the NanoDrop OneC spectrophotometer (Thermo Fisher Scientific). Equal amounts of lysates were resolved by SDS–PAGE and analyzed by immunoblotting with the indicated antibodies.

For IP of tagged proteins, ∼1 mg of lysate was incubated for 3 to 4 h at 4 °C with agarose beads conjugated to tag-specific antibodies. Following incubation, immunocomplexes were washed three times with NETN buffer (20 mM Tris-HCl, pH 8.0, 100 mM NaCl, 1 mM EDTA, and 0.5% NP-40), resolved by SDS–PAGE, and analyzed by immunoblotting.

### In-cell ubiquitination assays

HEK293T cells were transfected with His-Ub (WT or K48-linkage-only), along with other indicated constructs. Cells were treated with 10 μM MG132 before cell harvest. Cells were collected and lysed in buffer A (6 M guanidine-HCl, 0.1 M Na_2_HPO_4_/NaH_2_PO_4_, and 10 mM imidazole, pH 8.0) and then sonicated. Lysates were spun down at 12,000 g, and supernatants were transferred into a new 1.5 ml tube and incubated with Ni-NTA agarose beads for 3 h at room temperature. After incubation, beads were sequentially washed 3 times with buffer TI (25 mM Tris-HCl, 20 mM imidazole, pH 6.8). Bound proteins were resolved by sodium dodecyl sulfate-polyacrylamide gel electrophoresis and analyzed by immunoblotting with the indicated antibodies.

### ISD90 treatment

Cells were split into 6-well plates 1 day before treatment to ensure the cell confluence reached 70% to 80%. 5 μg/ml IDS90 was added into DMEM-only medium with 20 μl polyethylenimine and vortexed. After 15 min of incubation at room temperature, 800 μl DMEM with 10% FBS medium was added to form a 1 ml solution. Previous medium was removed from cells and replaced with the new 1 ml medium. At indicated time points, cells were harvested and lysed in EBC buffer. Equal amounts of cell lysates were resolved by SDS–PAGE and analyzed by immunoblotting with the indicated antibodies.

### RNA extraction and quantitative real-time PCR

Total RNA was extracted using the RNA Miniprep Super Kit (BS584, Biobasic) following the instruction manual. RNA concentration and purity were assessed with the NanoDrop OneC spectrophotometer (Thermo Fisher Scientific). cDNA was synthesized using the iScript cDNA Synthesis Kit (1,708,890, Bio-Rad) following the instruction manual. Quantitative real-time PCR was conducted using iTaq Universal SYBR Green Supermix (1,725,120, Bio-Rad) with the QuantStudio 6 Flex Real-Time PCR System (Thermo Fisher Scientific). Relative mRNA expression levels were normalized to U6 snRNA using the comparative Ct method. Statistical significance was evaluated using t tests and one-way ANOVA. Quantitative real-time PCR primers are listed below:

qRT-U6-F: CTCGCTTCGGCAGCACAT

qRT-U6-R: TTTGCGTGTCATCCTTGC

qRT-OTUD3-F: TGCGAGGAGGAGTTCGTCA

qRT-OTUD3-R: GTGCTTGAGATGATTTCGTGAGT

qRT-RSK3-F: GAAGAAGGCGTCGTGAAGGAG

qRT-RSK3-R: CCGAACTTTTAGGGTGGCTTT.

### Statistical analysis

Statistical analyses were performed using the Graphpad Prism 8 Software (www.graphpad.com). *p*≤ 0.05 was considered statistically significant. The results are shown as means ± SD from at least two or three independent experiments as indicated in figure legends. Differences between control and experimental conditions were evaluated by a *t* test and one-way or two-way ANOVA.

## Data availability

All data supporting the findings in this study are available from the corresponding author upon reasonable request.

## Conflict of interest

The authors declare that they have no conflicts of interest with the contents of this article.
